# Bacterial Microbiota of Asthmatic Children and Preschool Wheezers’ Airways—What Do We Know?

**DOI:** 10.3390/microorganisms11051154

**Published:** 2023-04-28

**Authors:** Kamil Bar, Maja Litera-Bar, Barbara Sozańska

**Affiliations:** 1 1st Department and Clinic of Paediatrics, Allergology and Cardiology, Wroclaw Medical University, 50-367 Wroclaw, Poland; 2 University Clinical Hospital in Wroclaw, 50-556 Wroclaw, Poland

**Keywords:** asthma, children, wheezers, microbiome, wheeze, airways, bacteria

## Abstract

Asthma is the most chronic pulmonary disease in pediatric population, and its etiopathology still remains unclear. Both viruses and bacteria are suspected factors of disease development and are responsible for its exacerbation. Since the launch of The Human Microbiome Project, there has been an explosion of research on microbiota and its connection with various diseases. In our review, we have collected recent data about both upper- and lower-airway bacterial microbiota of asthmatic children. We have also included studies regarding preschool wheezers, since asthma diagnosis in children under 5 years of age remains challenging due to the lack of an objective tool. This paper indicates the need for further studies of microbiome and asthma, as in today’s knowledge, there is no particular bacterium that discriminates the asthmatics from the healthy peers and can be used as a potential biological factor in the disease prevalence and treatment.

## 1. Introduction

Asthma is the most chronic pulmonary disease in the pediatric population, with a rising prevalence in some countries [1]. The most common cause of the disease exacerbation is viral respiratory tract infections; bacterial impact on exacerbations remains unclear [2]. A meta-analysis of *Mycoplasma pneumoniae* infection has shown a significantly higher ratio of IgM antibodies among the asthmatics, compared to the controls, as well as in the acute asthmatics, compared to the stable asthma group [3]. Early colonization with potentially pathogenic bacteria leads to a higher risk of bronchiolitis and pneumonia in the future apart from a higher risk of asthma prevalence [4]. Patients with eosinophilic asthma are more susceptible to bacterial-pathogen respiratory infections; however, between infections, bacteria abundance is relatively low and is mostly presented by common types [5]. Nontypeable *Haemophilus influenzae* is a commensal bacterium frequently found in nasopharyngeal swabs of healthy adults; however, it is also one of the most abundant strains in the lower airways of neutrophilic asthmatics. Some evidence proves that infection in early life increases the risk of asthma in the future on an animal model [6]. Asthmatic children are more susceptible to invasive pneumococcal disease based on the current meta-analysis [7], with a higher probability of developing pneumonia, compared to peers without any risk factors [8].

Since the launch of the Human Microbiome Project, our knowledge about the connection between our body systems and microorganisms has begun to increase vastly, allowing for the isolation of certain dysbiosis patterns within diseases [9]. Human airways are inhabited by many different bacteria. The upper- and lower-airway bacterial composition remains very similar and differs in terms of concentrations, in advance of the upper respiratory tract; it can be justified by the hypothesis of the lower airway colonization through droplets aspiration [10]. Various samples collected during bronchoscopy proved that the lower-airway bacterial microbiota in adults is a sum of the mouth and nasopharyngeal composition, acquired by subclinical aspiration [11]. The lower the tract of the airways, the lower the biomass of detected bacteria, whose composition is similar in each site among individuals [12]. Airway colonization starts from the first day of life and modulates the immune system [13]. Various environmental factors alter the composition of microbiota towards homeostasis or dysbiosis, which potentially leads to chronic illnesses [11].

The aim of this review was to highlight the current knowledge about respiratory tract bacteria among asthmatic children. We have also included preschool wheezers, since asthma diagnosis in children below 5 years of age remains challenging due to the lack of sufficient tools for its confirmation [14].

## 2. Upper-Airway Microbiome

As for the asthmatics, the most common sample types were nasal/nasopharyngeal swabs, followed by throat swabs, nasal washes, brushings, induced sputum, nasal blow and saliva (Table 1).

Nasal brushes were characterized by higher alpha and beta diversity and more abundant bacterial microbiome than nasal washes [17]. One study used saliva as examined material [25]. In Espuela-Ortiz et al.’s study, 114 participants aged 8–21 years were equally divided into the asthmatic and control groups [25]. The whole study population was African American with the inclusion criteria of all four grandparents being African American. Asthma was defined as physician-diagnosed with at least one of active disease symptoms 2 years prior to the study. All samples in 98% of reads were dominated by the phyla Firmicutes, Proteobacteria, Actinobacteria and Fusobacteria. When comparing the groups, alpha diversity in terms of species richness was higher among the asthmatics. The study group had a higher abundance of *Veilonella* and lower *Streptococcus* than that in healthy children. No relevant differences were found in the other most abundant genera found in the samples: *Haemophilus* and *Prevotella*. An et al. compared various upper and lower-airway samples (mouth/nose/throat swabs, induced sputum, bronchial fluid) [23]. Twenty children aged 5–16 years, of which seven were asthmatics with no specific criteria of disease diagnosis mentioned, underwent tonsillectomy. Before the procedure, induced sputum was obtained, and then after intubation, nasal secretions, mouth and pharyngeal swabs were collected. Lastly, a bronchial cytology brush was inserted through endotracheal tube for lower airway samples. From all sites, overall bacterial profiles of nasal and bronchial samples were distinct from the mouth, throat and induced sputum, which clustered together. The most dominant phyla in all sample types were Actinobacteria (most dominant in the nasal and mouth swabs), Firmicutes, Fusobacteria (most dominant in the throat samples) and Proteobacteria. Bacterial compositions did not differ statistically in each sample sites between asthmatics and non-asthmatics.

Two studies assessed nasal epithelial cells. A study on 14 children showed greater species richness and less evenness in terms of alpha diversity; asthmatics’ samples were dominated by *Moraxella* species [15]. Moreover, in this study, host gene expression was also assessed with the use of libraries from the Human Microbiome Project database. The *Moraxella*-dominant samples were associated with the response of 32 genes previously connected with the immune response to these bacteria, whereas the control group showed no response. Another study was conducted on 134 patients aged 4–8 years with chronic rhinosinusitis, with asthma being a differential factor among the groups [28]. Nasopharyngeal swabs were taken for microbial assessment, and nasal epithelium was also obtained for host gene expression, as in the previously mentioned research. The asthmatic group was characterized by reduced alpha diversity (Shannon index) and an increased abundance of Actinobacteria and *Staphylococcus*. Moreover, asthmatic children were found to consume more sweets. Nasal washes were used in three consecutive studies conducted by Perez-Losada et al. [17,18,22]. In all of the following papers regarding this researcher, the study groups were asthmatic children of the AsthMaP-2 study. All children, aged 6–18, had been diagnosed with the disease at least one year prior to enrollment, most of the group’s ethnicity was African American. The exclusion criteria were a chronic or complex cardiorespiratory disease. In the first study, nasal washes of 40 asthmatic children were for bacterial composition taken during the first visit and another wash about 6 months later (±0.5 month) [18]. No relevant changes were observed in terms of alpha and beta diversity; however, the proportions of *Haemophilus*, *Moraxella*, *Staphylococcus* and *Corynebacterium* genera varied between sample collection times. The *Moraxella* genus accounted for more than 1/3 of reads (35.3%). An interesting finding in this particular study was that from all of the most abundant genera mentioned above, only *Haemophilus* had a seasonal difference in a relative proportion, with a higher abundance in the summer, compared to fall. The latter study conducted by the same head researcher with the same sampling method enrolled 163 children aged 6–18 years, of whom 42 attended a follow-up procedure after about 6 months (±0.5 month) and had another sample taken [22]. Within the study, three main asthma phenotypic clusters of patients were established based on various data acquired from the participants. The first cluster, APC 1, was female-dominant, with a lower asthma-control test score. The APC 2 cluster was characterized by the highest positive allergen-test ratio with the highest blood eosinophil rate and serum IgE value. Lastly, the APC 3 patients had the highest mean asthma-control test score and best outcomes of post-bronchodilator pulmonary test functions. When it comes to the most dominant phyla within the samples, the most abundant were Firmicutes, Proteobacteria, Actinobacteria, Bacteroidetes and Fusobacteria. The core microbiome genera presented in at least 95% of samples were *Moraxella*, *Streptococcus*, *Staphylococcus* and *Haemophilus*. Each mentioned cluster had its own significant variations in nasopharyngeal microbiome. APC1 was presented by the highest abundance of Actinobacteria and Bacteroidetes, with the dominant genera of *Corynebacterium* and *Prevotella*. APC2 participants had the highest abundance of Proteobacteria and Moraxella. The APC3 cluster was characterized by a higher abundance of Firmicutes and Fusobacteria, whereas at the genus level, no statistical differences were found, compared to other groups. Perez-Losada also compared nasal washes with nasal brushes of 30 asthmatic children [17]. The most abundant genera in nasal washes were *Moraxella* and *Staphylococcus*, whereas the most abundant in nasal brushes were *Moraxella* and *Corynebacterium*. Both sample types were different in terms of beta biodiversity and alpha biodiversity, with nasal brushes being enriched with more different bacteria. Moreover, in terms of core microbiome analysis (OTUs detected in at least 95% of samples), the main genera in nasal washes/washes OTUs were Moraxella, Pseudomonas, Enterococcus and Bacteroidetes and in nasal brushes/brushes OTUs of the genera Moraxella, Staphylococcus, Haemophilus, Streptococcus and Enterobacter. Another prospective study was meant to answer if there are any bacterial changes in the early loss of disease control [24]. A total of 254 children aged 5–11 years with doctor-diagnosed asthma of mild to moderate persistence and at least one exacerbation in the previous year were treated with a low dose of fluticasone propionate (twice 88 ug daily), with a randomization of taking either the same or a five times higher dose on the early signs of loss of asthma control (“yellow zone” defined as the use of certain amounts of rescue albuterol in a day and any night awakening due to asthma with the need of this medication); they were originally a part of another study. Thirty-eight nasal blows were taken by a researcher during the randomization visit, followed by the second blow taken at home during the early stage of disease-control loss by parents who were previously thought of the sampling technique. Samples of younger children aged 5–7 years during the randomization visit had a higher abundance of *Moraxella* and *Streptococcus*; they were more at a higher risk of developing symptoms of early asthma-control loss than older participants. Older children had a higher abundance of *Staphylococcus*. Taken the fact that viral infections are associated with asthma exacerbations, the researchers also assessed viral genetic material within the samples, of which mostly rhinoviruses were detected. Viral-positive samples (33% of randomization samples) were associated with a higher abundance of *Moraxella* and a lower abundance of *Bacillus* and *Staphyloccoccus*. Using clustering analysis, the dominant genera were obtained, of which *Corynebacterium + Dolosigranulum* lowered the risk of the annual rate of “the yellow zone” symptoms and elongated the time between episodes in children, who reported at least two of them in one year. During the yellow zone sampling, more than half of the patients switched their dominant cluster to the *Streptococcus* cluster, which became most dominant at this point of the study. Moreover, total bacterial load and species richness were also higher at the early disease-control loss stage. Switching from the *Corynebacterium + Dolosigranulum* cluster at randomization to the *Moraxella* cluster at early symptoms of losing control was associated with the highest risk of progressing to asthma exacerbation. In Kim et al.’s study, nasopharyngeal swabs were taken from the children of three groups: asthma (*n* = 31), asthma in remission (*n* = 30) and healthy controls (*n* = 31) [21]. Children who participated were a part of KOREA study, the specimens taken in this particular study were from children aged 6–10 years. Asthma was diagnosed by pediatric allergologist on both clinical symptoms and either with positive methacholine challenge test (MCT) or at least 12% of forced expiratory volume improvement after albuterol administration, according to the American Thoracic Society guidelines. Asthma remission was defined as no symptoms and no need of asthma medication for at least 2 years prior to enrollment, with additional normal MCT results. All children reported no antibiotic therapy 3 months prior to sampling and at least 2 months of neither intranasal nor inhaled corticosteroids. At the phylum level, Proteobacteria, Firmicutes and Fusobacteria were most dominant, with a statistically higher proportion of Proteobacteria among the control group, Firmicutes among the asthmatics and Fusobacteria in remission. at the genus level, *Staphylococcus* was most abundant in the asthma group, whereas *Haemophilus* and *Moraxella* were more abundant in the control group. Both asthmatic groups active and in remission had a higher abundance of *Streptococcus*, *Dolosigranulum* and *Corynebacterium* than that in the healthy group. Additionally, in the remission group, the abundance of Fusobacterium, *Prevotella* and *Parvimonas* was the highest. Firmicutes at the phylum level and *Staphylococcus* at the genus level were inversely associated with the concentration of methacholine in the MCT test. Moreover, the Streptococcus genus abundance was negatively associated with the predicted FEV1. Within the study, functional genes were also obtained from the samples. When comparing taxonomical findings, Streptococcus pneumoniae in the asthmatics were associated with arachidonic acid metabolism genes, which were more abundant in both asthmatic groups. Genes associated with lysin degradation were less abundant in the asthma group than in remission group. The *Haemophilus influenzae* species was associated with these lysin degradation genes in all groups, whereas for the asthmatic group, only specific were *Neisseria lactamica*, *Neisseria meningitides*, *Parvimonas micra* and *Treponema medium*.

In Hou et al.’s study, the change in the asthmatics’ bacterial microbiota during the disease exacerbation was assessed [30]. Thirty-three asthmatics aged 6–17 with physician-diagnosed asthma and at least one asthma exacerbation within the past 12 months were enrolled, as well as 20 non-asthmatic children as the control group. Asthma exacerbation was defined as either more than three uses of a beta agonist in at least 2 consecutive days, use of oral prednisolone or an unscheduled physician visit or hospitalization regarding asthma. Flocked nasopharyngeal swabs were obtained from both groups of participants, as well as five more during follow-up home visits at 2–4-week intervals. Similar to other studies, the most abundant phylum was Fimicutes, followed by Proteobacteria, Actinobacteria, Bacteroidetes and Fusobacteria. Researchers distinguished six nasopharyngeal microbiomes by most abundant genera within *Moraxella*, *Corynebacterium 1*, *Dolosigranulum*, *Staphylococcus*, *Streptococcus* and *Anoxybacillus*. A higher proportion of the *Corynebacterium-1*-oriented cluster was observed in the stable asthma group, and none was detected in the asthma exacerbation group. During asthma exacerbation, the alpha diversity in the samples lowered substantially. Interestingly, the *Moraxella* abundance almost doubled at the exacerbation point, whereas *Corynebacterium 1* and *Dolosigranulum* showed a significant decrease. Moreover, a *Moraxella* increase during the exacerbation contributed to nicotinate and nicotinamide metabolism. Liu et al. also tried to assess microbial changes during exacerbation [31]. Fifty-six asthmatic children aged 3–17 years with no control group were recruited during asthma exacerbation defined as a progressive worsening of symptoms and the need of reliever medications. The endpoint group were patients in a “recovery phase” approximately 2 weeks after exacerbation. The exclusion criteria were fever during a worsening, antibiotic therapy, an oral or parenteral steroid course for over 15 days, use of high budesonide doses in inhalation and need for at least four doses of β2-agonist a day. Patients had their nasopharyngeal swabs, throat swabs as well as stool samples collected. During the recovery phase, eight microbial clusters were distinguished, with *Corynebacterium + Dolosigranulum* and *Staphylococcus* being the most frequently found in the nasopharyngeal samples. Children of 3–5-year-old samples showed a statistically relevant change between the two time points. Moreover, moving towards the recovery phase, the *Staphylococcus* abundance increased while that of *Moraxella* and *Acinetobacter* decreased. The *Corynebacterium + Dolosigranulum* cluster was also positively associated with IgE levels in serum. Throat samples did not alter during the researched time phases, indicating no association with asthma. Researchers also compared bacterial composition of the asthmatics to other respiratory diseases. Boutin et al. compared throat swab samples of patients with cystic fibrosis (CF), asthmatics and healthy peers; all of participants were aged 6–12 years [19]. CF patients (*n* = 57) were diagnosed by specialists. Data on the asthmatic children (*n* = 27) were obtained from GABRIELA study, and asthma was defined using one of the three criteria: a parent reporting a wheeze at least two times in 12 months, a positive answer to the question about the “asthma spray” use or doctor-diagnosed asthma or a least two or more instances of obstructive bronchitis. The control group consisted of 62 healthy children. Pediatric cystic fibrosis patients had a lower *Haemophilus* abundance and less diverse bacterial microbiota than the asthmatic children. In the asthmatic group, the absence of one OTU belonging to *Aggregatibacter* was found and compared to other cohorts. When comparing the swab samples of the asthmatics and healthy controls, no difference in alpha diversity was found. Aydin et al. connected the rhinobiome of asthmatics with recurrent wheezers and healthy controls [29]. The alpha diversity in the nasopharyngeal swabs of atopic asthmatics increased, compared to a decrease in the atopic wheezers. Proteobacteria were more abundant in the wheezers group, while Actinobacteria were more abundant in the healthy controls and Firmicutes in the asthmatics. *Moraxella* was found to be the most abundant within the wheezers group, as well as the *Haemophilus* genus. The nasal cavity samples of atopic asthmatics had a higher abundance of Streptococcus and Staphylococcus. Interestingly, in this study, nasal epithelial spheroid cultures of healthy controls were infected with the *Moraxella catarrhalis* strains of the asthmatics and wheezers groups, leading to disruption of epithelial integrity after 24 h of incubation.

Environmental factors also seem to have an effect on asthma microbiota. Birzele et al. compared mattress dust samples and nasal swabs of children living on farms with non-farm residents, as a part of GABRIELA study [20]. One hundred and two children aged 6–12 years were enrolled. Asthma definition was the same as mentioned above regarding GABRIELA project. Non-asthmatics’ nasal and mattress samples were characterized by a higher richness of bacteria than in the asthmatics. Mattress-dust bacterial richness was found to be a protective factor in asthma prevalence. Alpha diversity of mattress-dust bacterial microbiota was higher among the farm children, whereas the nasal samples’ higher diversity was associated with the exposure to cow and straw. The *Prevotella* abundance in the nasal swabs was inversely associated with asthma. Interestingly, when comparing all nasal samples regardless of the group, farm exposure did not alter biodiversity significantly. Another related study by the GABRIELA working group included 327 throat samples and 68 nasal swabs from children aged 6–12 years with asthma definition as mentioned previously [16]. In both sample types, the asthma status nor farm exposure did not alter the bacterial load. In beta diversity of only nasal samples, the greater phylogenetic similarity was found among the asthmatics. Alpha diversity in the nasal simples of the asthmatic children was significantly lower than that in the non-asthmatic peers, with no change, compared to the farm-exposure group. Given the taxonomics, the bacterial relative abundance in the nasal samples of the asthmatic children showed a higher Proteobacteria load at the phylum level and *Moraxella*, a member of the phylum, at the genus level. The specific abundance of *Motraxella OTU 1462* was inversely correlated with the nasal bacteria richness, but only for non-farm children. Besides rural animal exposure, another research study investigated house pet exposure, sensitization and microbiota [27]. The study enrolled 132 children with persistent asthma, with a mean age of 12 years, with asthma diagnosed using the National Heart, Lung and Blood Institute Expert Panel Report 3 Guidelines, taking various asthma medications on a daily basis and with SABA predominance (92%). Specific IgE higher than 0.1 kU/l was treated as sensitization to cat (68.9% participants) and dog (72.7%) allergens. Nasal swabs and brushing of participants revealed significantly lower alpha and beta diversity among the asthmatics sensitized to cat allergens, whereas there was no statistical difference in terms of dog sensitization. The *Corynebacterium* spp. And the *Staphylococcus epidermidis* abundance was associated with the absence of sensitization to cat or/and dog allergens. Additionally, this association was related to reduced 7-gene expressions related to IgE-mediated hypersensitivity and mast cell function. Other studies investigated if airway allergies have an impact on the microbiome. In Chiu et al.’s study, 60 children aged 4–5 years were enrolled, of which 38 were mite-sensitized and 22 were healthy controls [26]. From the study group, 18 children suffered from asthma and the other children from allergic rhinitis, both diagnoses were physician-diagnosed by a pediatric pulmonologist. All patients had not received antibiotic therapy 4 weeks prior to enrollment. Sensitization to mite dust was assessed by specific IgE levels of at least 17.5 kU/L (class 4 and above). From all participants, throat swabs and stool samples were obtained. The most abundant phylum in throat samples was Firmicutes, followed by Bacteroidetes, Proteobacteria and Fusobacteria. At the genus level, the most common were *Streptococcus*, *Prevotella* and *Fusobacterium*. As expected, lower diversity in terms of richness was observed in the airway samples, compared to the stool samples. Moreover, the alpha diversity richness in the throat samples was lower among children with mite-sensitization, but the main significance was found in children with rhinitis and not asthma, compared to the healthy group. The asthmatic children had a significantly higher abundance of *Leptotrichia* and *Selomonas* in the airway samples than patients with rhinitis. An interesting finding of the study is that the airway microbiota correlated mostly with total fecal IgE and not serum levels. Total fecal IgE levels were positively correlated with specific IgE of *Dermatophagoides pteronysinnus* and *Dermatophagoides farinae*, as well as the *Haemophilus* abundance in the airways. Contrary, a negative correlation of bacterial abundance to stool IgE was found for *Atopobium*, *Bulleidia*, *Moryella* and *Dialister*.

As for recurrent wheezers solely, the presented studies’ samples were nasal and oropharyngeal swabs. All of the papers described prospective studies (Table 2).

Powell et al.’s prospective research study enrolled healthy term newborns, observed up to 24 months of age [32]. Oropharyngeal swabs from the posterior wall of the pharynx were obtained during home visits at 6 weeks, 6–9–12–18 and 24 months of age. After 2 years of visits, medical history of patients was obtained from their general practitioners to search for wheezing episodes. All necessary data and samples were obtained for 98 patients. Doctor-diagnosed wheeze was defined as recorded in medical notes, auscultation-confirmed or with a prescribed bronchodilator. Recurrent wheeze was defined as at least two wheeze episodes. Twenty-six children were diagnosed with wheeze, of whom eleven had recurrent wheezing. The bacterial density of children observed from birth to 24 months increased significantly at up to 9 months of age. Alpha and beta diversity of microbiota were correlated positively with age, with the highest change between samples taken at 6 weeks of age and any other assessment. Children with a wheeze diagnosis had a higher abundance of the *Neisseria* OTU, whether non-wheezers samples were enriched more with the *Granulicatella* and *Prevotella* OTUs, with *Neisseria* starting to differ after 9 to 24 months of age. Interestingly, at 6 weeks of age, all of the three OTUs were equally abundant. Another study conducted by Cuthberston et al. compared bacterial microbiota of children 0–16 years of age with acute wheeze and their healthy peers [33]. Oropharyngeal swabs of 109 acute wheezers (median age 3.8 years) and 75 non-wheezing children (median age 3.1 years) were obtained; in addition, follow-up samples were collected from 17 children from the wheezing group. Alpha diversity between the groups showed no statistically relevant differences. Diagnosis of bronchiolitis was a factor decreasing alpha diversity. Assessing only the acute wheeze group, the researchers also found that kindergarten attendance increased bacterial richness of oropharyngeal samples. The follow-up samples did not differ in alpha diversity either. However, beta diversity indicated the presence of distinct communities.

In Mansbach et al.’s study, 842 infants (median age 3 months) hospitalized with the first severe bronchiolitis were followed up to the age of 3 years for recurrent wheezing [35]. Each child had their nasal swab taken by a specialist in a hospital, and then parents after proper instruction collected another sample 3 weeks after hospitalization, in the summer while the children were healthy and 1 year post-hospitalization. In this multicenter study (35th Multicenter Airway Research collaboration, MARC-35), attending pediatricians diagnosed bronchiolitis based on the American Academy of Pediatrics’ definition. Recurrent wheezing was defined by the 2007 National Institutes of Health Expert Panel Report 3—at least two corticosteroid-treated exacerbations in 6 months or at least four wheezing episodes in a year that lasted 1 week and affected sleep. Additionally, they extended the endpoint to the age of 4 years and asthma diagnosis, which was defined as physician-diagnosed with either asthma medication usage or specific for its symptoms. The collected data identified that an increased abundance of *Moraxella* and *Streptococcus* 3 weeks after hospitalization or of *Streptococcus* in the summer after hospitalization was associated with a higher risk of recurrent wheezes by the age of 3 years, highlighting the possibility of post-illness colonization intervention to prevent the disease. Moreover, the same results in terms of developing asthma at the age of 4 years were obtained, with the exclusion of a higher abundance of *Streptococcus* in the summer samples, which did not increase the risk. The same cohort of MARC-35 was used in a study conducted by Dumas et al. [34]. The difference of this prospective study was that the only samples were taken during hospitalization, and the follow-up was the telephone interviews with parents of the children 1 week after hospitalization, 3 weeks after and then every 6 months from the age of 6 months onwards. Nine hundred and twenty-one children were clustered into three profiles (from A to C) depending on the latent class analysis. Profile A had the highest proportion of children with *Moraxella*-dominant profile and had a higher proportion of *Haemophilus* profile as well, compared to profile B. These children had a higher rate of rhinovirus infection and a higher proportion of eczema and breathing problems. Profile C was characterized as well by more children with *Haemophilus*-dominant profile than B, but the lowest proportion of *Moraxella*-dominant among all groups. The C group was mostly infected with RSV during enrollment hospitalization and presented more severe respiratory distress symptoms, as well as 21% of them being hospitalized for more than a week, in contrast with 0 and 1% in the other groups. In another study by Tang et al., a cohort of 289 newborns were enrolled for a 3-year follow-up [36]. All children were in risk of atopic diseases having at least one parent with asthma or other allergic diseases. Nasopharyngeal samples were obtained at 2, 4, 6, 9, 12, 18 and 24 months of age. Additional samples were taken during upper respiratory tract disease of at least moderate severity and any lower-airway infection up to 3 years of age. Wheezing respiratory illness was defined in the previous study with the same cohort as physician-diagnosed wheezing, illness with prescribed beta agonist and/or long-term controller medication or illness with diagnoses of bronchiolitis, wheezing illness, reactive airway disease, asthma or asthma exacerbation. All participants were divided into four trajectory groups of microbiome composition with each being represented by a distinct bacterial taxon in the first 4–6 months of life. The trajectory dominated by *Staphylococcus* was associated with a higher prevalence of wheezing illnesses of the second and third year of life. The acquired data allowed the researchers to extract four clusters associated with acute respiratory illnesses. Bacteria of discriminant clusters were the OTUs of *M. catarrhalis*, *S. pneumoniae* and two of *H. influenzae*. Interestingly, in this study, viruses were also taken into account. During illnesses of the respiratory tract, the most dominant correlation was in the detection of both viral and above-mentioned pathogens (66% of cases) than each individually. Moreover, the *Moraxella* dominance during acute wheezing was associated with asthma persisting through later childhood. The aspect of recurrent wheezing and infections was also assessed in the study conducted by Song et al. by the use of nasopharyngeal swabs [37]. Children aged 2–5 years were divided into the recurrent wheezing group (*n* = 16), who were hospitalized because of this diagnosis and had a positive stringent-asthma predictive index, the inpatient control group (*n* = 18) of children with no history of asthma or wheezing but having symptoms of upper respiratory tract infection by the enrollment time and the community control group (*n* = 36) with no allergic diseases, free of respiratory tract infection symptoms for at least 4 weeks prior to sampling. The additional exclusion criterion was antibiotic treatment in 4 weeks before the study. All samples were checked for the human rhinovirus presence. In all of the recurrent wheezers, the genetic material was found, whereas in the other groups, less than a third of participants were positive for that virus. *Moraxella catarrhalis* and *Dolosigranulum pigrum* were the most abundant species in all samples. The only phylum discriminant for recurrent wheezers was Proteobacteria with no particular genus of species of statistically higher dominance. A higher abundance of *Dolosigranulum pigrum* was found in the community control group, when it was adjusted for the human rhinovirus status. Interestingly, the *Haemophilus influenzae* abundance was higher in inpatient controls with respiratory tract infection, and it was primarily discriminant in samples positive for the rhinovirus within the group.

Bacterial upper-airway microbiota seems to be consistent at the phylum level regardless of asthma and wheezing, with Firmicutes, Proteobacteria, Actinobacteria and Fusobacteria being most abundant. At the genus level, the main differences concern pathogens such as *Moraxella*, *Haemophilus*, *Staphylococcus* and *Streptococcus*, which dominate in specific clusters and affect the niche in terms of biodiversity and transcriptome, which potentially may be a key to a better understanding of improper immunological responses in asthma and other allergic diseases.

## 3. Lower-Airway Microbiome

Data about bacterial microbiota of the lower airways are much more limited than those of the upper part, and the study participants were much fewer (see Table 3 for asthmatics and Table 4 for wheezers). Besides induced sputum, the only noninvasive method within the assessed papers, other sample types were bronchoalveolar lavage (BAL) and bronchial brushing. Additionally, in one study, sputum was collected from the trachea by a soft catheter [38]. Induced sputum however involves a risk of contamination from the upper respiratory tract and is not recommended by some researchers in children because of insufficient susceptibility, compared to other methods [23].

One of the first studies regarding children’s lung microbiota was conducted by Hilty et al. [39]. Bronchoalveolar lavage fluid was collected from a small group of 13 asthmatic children with difficult asthma, characterized by the need of treatment with a high dose of inhaled corticosteroids and long-acting β2-agonists and/or oral prednisolone, as well as using rescue bronchodilator at least three times a week; seven controls were also included for comparison. The study revealed a higher abundance of Proteobacteria at the phylum level. At the genus level, a higher abundance of *Haemophilus* and *Staphylococcus* was observed, compared to the non-asthmatic control group. Accordingly, non-asthmatics were characterized by a higher abundance of Bacteroidetes and *Prevotella*. The same study assessed the adults’ lower airways with bronchial brushing of the left upper lobe, and the bacterial outcomes of both studied groups regardless of age were similar [39]. Limitations of the study were a small group of pediatric patients and a lack of a healthy control group; non-asthmatic children that underwent bronchoscopy had other comorbid diseases. Goldman et al. also assessed the lower airways’ microbiota with bronchoalveolar lavage [41]. Thirty-one study participants were divided into severe asthmatics (*n* = 15, mean age 11 years), cystic fibrosis patients (*n* = 5, mean age 14 years) and the non-asthmatic group (*n* = 11, mean age 5 years). Children with severe asthma were defined as treated with high-dose inhaled or oral corticosteroids for at least half a year, with at least two minor additional criteria present. All were treated with a combination of high-dose inhaled corticosteroids and long-acting beta agonists, with or without other asthma medications. Six children also were sensitized to fungi. The non-asthmatic group included children with congenital respiratory malformations, e.g., tracheal diverticulum, tracheomalacia, etc. At the phylum level, the most dominant taxa were Firmicutes, Proteobacteria and Bacteroidetes in all groups. In all of the samples, the most dominant genera were *Streptococcus* and *Prevotella*. Regarding the asthmatic patients, 10 genera were discriminant, compared to the non-asthmatics, with a higher abundance of *Bacteroides*, *Faecalibacterium* and *Roseburia*. Moreover, a higher abundance of *Proteus* and *Capnocytophaga* was found among the non-asthmatics, compared to the asthma patients. The authors also indicated that the non-asthmatic group was younger than the others and had evidence of inflammation in the samples, which could influence the results. Some researchers tried to compare lower microbiota findings with those for the upper airways. Kloepfer et al. compared bronchoalveolar lavage fluid (BALF) samples with nasopharyngeal swabs [40]. Thirty-six children aged 3 months–18 years that underwent bronchoscopy had various diagnoses, in which asthma affected 61% of patients and recurrent bronchitis/pneumonia 67%. There were no specific criteria mentioned in the text for defining diseases that were the reasons for the procedure. Four patients received antibiotic therapy during enrollment, whereas others claimed to be withdrawn from any at least 2 weeks prior to sampling. Twenty-four bronchial fluid samples (66.7%) were obtained from at least two or three lobes. The BALF samples were found to be more diverse and richer in bacteria than swabs with the use of alpha and beta diversity. Both sample types were dominated by Firmicutes and Proteobacteria in relative abundance. Nasopharyngeal swabs had a higher abundance of Actinobacteria, whereas BALF was characterized by a higher abundance of Bacteroidetes, compared to each other. At the genus level, relevant differences in advance of nasopharyngeal swabs regarded *Corynebacterium*, *Staphylococcus*, *Moraxella* and *Haemophilus*, whereas the lower airways had a higher abundance of three *Prevotella*-family genera. The *Streptococcus* genus in both sample types was the most dominant. BALF samples of conventional bacterial cultures were also obtained; in 22 of 34 samples, at least one or more bacteria were culture-positive. When comparing to the assessed 16S rRNA, all culture-positive bacteria were also present in the molecular outcomes, with no correlation between the OTU abundance and its presence in the standard culture. Chun et al. also compared nasal swabs with BAL fluid samples of the asthmatic children and the control group to assess bacterial microbiome and transcriptome [42]. The groups consisted of 27 children with severe persistent asthma and the same amount of healthy controls with a mean age of 12 years (~4 years SD). There was no clear definition of severe persistent asthma in the study. Nasal swabs and BAL fluid samples were obtained for a microbiome assessment, and nasal and bronchial brushings for a transcriptome. However, due to invasive procedure, in this particular study, only asthmatic patients underwent bronchoscopy and had their lower airways’ samples collected. In the asthmatics group, nasal and BAL fluid samples varied in terms of both alpha and beta diversity, with bronchial samples having higher species richness. *Corynebacterium*, *Staphylococcus* and *Moraxella* were more abundant in nasal samples, while *Veilonella*, *Prevotella*, *Streptococcus* and *Neisseria* dominated in bronchial samples. Researchers also assessed the network of the associations between each genus. In nasal microbiome of the asthmatics, *Moraxella* and *Alloiococcus* were hub genera, with none of them in the BAL fluid samples. Taking into account the networks between the nasal transcriptome and microbiome, the study showed that *Corynebacterium* represented 30% of the associations, and all of them were negative, leading to lower gene expression with its higher abundance. However, that connection did not lead to any enrichment of biological processes. Interestingly, the same hub genera were found among the healthy peers, where *Corynebacterium* was negatively correlated with inflammation-promoting genes, and suggest their potential protective role in the non-asthmatics. Moreover, *Actinomyces* in the lower airways were negatively correlated with inflammation genes and can potentially be a protective factor. Kim et al. compared the induced-sputum microbiota of asthmatic children during exacerbation, with that of the stable asthma group and healthy peers, assessing the bacterial composition and its connection with inflammation cytokines [43]. Ninety-five children aged 6–15 were enrolled in the study. Asthma was diagnosed based on the current respiratory symptoms and confirmed in spirometric tests according to the ATS guidelines. Stable asthma was defined as no exacerbation in the last 4 weeks with the need of a systemic corticosteroid or an increased amount of inhaled corticosteroids, use of rescue medication less than 3 times a week and no need for a medication change. Asthma exacerbation was a disease worsening that required systemic steroid usage or hospitalization. Among 22 children in the exacerbation group, 11 were diagnosed with RSV concomitant infection, and one had a positive result for an influenza virus in the PCR analysis of nasopharyngeal swabs. No relevant differences in alpha diversity were found among the stable asthma and exacerbation groups; however, beta diversity showed statistical significance in terms of the dissimilarity of the two communities. Proteobacteria were more abundant, and Actinobacteria were less abundant in the asthma exacerbation group. At the genus level, the abundance of *Haemophilus*, *Campylobacter*, *Neisseria*, *Veilonella* was higher in the same group of patients. Moreover, in the exacerbation group, a microbiota network was generated to assess their shared correlation, and the mostly connected genera were *Campylobacter*, *Haemophilus*, *Neisseria*, *Fusobacterium*, *Streptococcus*, *Peptostreptococcus* and *Granulicatella*. In the same study, inflammation cytokines were also assessed from the sputum. *Campylobacter* was found to be positively correlated with several inflammatory cytokines, such as granzyme B, macrophage inflammatory protein 1β (MIP-1β) and programmed death-ligand 1 (PD-L1). Additionally, *Haemophilus* was positively correlated with PD-L1, while *Porphyromonas* and *Peptostreptococcus* had negative correlation. The researchers also conducted the original research of the lower-airway microbiota using the exhaled breath condensates, which to our knowledge was the first study in pediatric asthmatics [44]. Exhaled breath condensates (EBCs) and oropharyngeal swabs were obtained from 38 children (19 asthmatics vs. 19 healthy participants). The exclusion criteria consisted in no antibiotic treatment and any respiratory illnesses at least 30 days prior to sampling. Asthma was defined as being diagnosed in the past by an allergologist. The most abundant phyla of both the lower and upper airways were Firmicutes, Proteobacteria and Actinobacteria. Between the asthmatics and the control group’s samples of the exhaled breath condensates, the class Gammaproteobacteria and genus Bacilli were more abundant in the asthmatic patients. In the comparison of the samples, the swabs were characterized by a higher abundance of bacterial species than the EBCs. In terms of biodiversity, the asthmatics’ lower-airway samples had higher bacterial species diversity (Shannon diversity index) and more even distribution (Pielou’s evenness), than those in the healthy children.

As for wheezers, the lower-airway bacterial findings were similar to those of the asthmatics.

Robinson et al.’s study concerned episodic viral and multiple-trigger wheezers [45]. Thirty-five children aged 1–6 years with severe recurrent wheeze underwent bronchoscopy during infection with BAL fluid sampling. Wheeze was doctor-diagnosed and confirmed using a video questionnaire. The study groups were divided via a parental questionnaire into episodic wheezers during viral upper-respiratory-tract infections and multi-trigger wheezers with additional wheezes between infections. Based on the standard bacterial culture and viral detection tests, 60% of children had a positive outcome on either test. The most common bacterial results were *Streptococcus pneumoniae*, *Haemophilus influenzae*, *Moraxella catarrhalis* and *Staphylococcus aureus*. A higher neutrophil count of BAL fluid was found in multi-trigger wheezers with positive bacterial cultures. Due to other clinically indicated tests, BAL samples from only 26 patients could be assessed for the microbiome analysis, and hence, some of the samples collected were insufficient in the latter aspect of 16S amplification and sequencing; bacterial OTUs were acquired from only 14 patients. Although no significant differences between two study groups have been found in terms of microbiota, taking all participants (into account) two other groups have been observed, with Moraxella genus being discriminant. The *Moraxella* species profile was associated with higher neutrophil counts in BAL fluid samples, while the other group of a mixed-microbiota cluster was characterized by a higher concentration of BAL macrophages and lymphocytes. All wheezers with this particular profile had Moraxella-catarrhalis-positive fluid culture outcome. The other group called “mixed” was in contrast characterized by higher bacterial diversity. Another study conducted by Wu et al., compared BAL samples of 35 infantile persistent wheezers up to 2 years old and a control group of 28 peers with foreign-body aspiration that underwent bronchoscopy [46]. Persistent wheezing was assessed according to the ATS recommendations, as the duration of a wheezing episode of more than one month despite proper treatment was an indication for further diagnostics with the use of bronchoscopy. All wheezers were followed up 24 months after enrollment to assess the recurrence of symptoms; more than half (*n* = 20, 57.1%) had at least one episode of wheezing, and they were further described as the “recurrence” group. No differences were measured using alpha diversity between the groups, although wheezers’ samples for relative abundance were more enriched with *Elizabethkingia* and *Rothia* and had lower concentrations of *Moraxella* and *Fusobacterium* at the genus level. The most dominant phyla detected in both groups were Proteobacteria, Firmicutes, Bacteroidetes and Actinobacteria. In the wheezer group solely, boys had a lower abundance of *Haemophilus*, *Prevotella* and *Porphyromonas*. Moreover, the route of delivery was a discriminating factor in the cesarean-section group with a lower abundance of *Streptococcus*, *Alloprevotella* and *Prevotella* and a higher abundance of *Sphingomonas*, *Elizabethkingia* and *Phenylobacterium*. Children of the “recurrence” group at the genus level had also a higher abundance of *Elizabethkingia* and *Rothia,* compared to the wheezers with no wheezing episodes in a 2-year follow-up. Using sputum samples taken from the trachea, Zhang X. et al. conducted a follow-up study of infants with the first severe RSV bronchiolitis, assessing microbiota and whether they develop recurrent wheezing by the age of 3 years [38]. The initial age of the children was less than 6 months; 74 patients were included. The exclusion criteria were any chronic illness, concomitant infection caused by other pathogens, any wheezing in the past, use of anti-gastroesophageal reflux medication and prematurity. Wheezing in a follow-up was diagnosed as a yes answer to the question about a wheezing episode requiring corticosteroid inhalation. Similar to other studies, the most dominant phyla were Firmicutes, Proteobacteria, Bacteroidetes and Actinobacteria. At the genus level, *Streptococcus* was highly dominant above all with an 80% relative abundance, with *Haemophilus* and *Moraxella* as the top three most frequently isolated genera. Infants who were later on diagnosed with recurrent wheezing (defined as three or more wheezing episodes, *n* = 26; 35.1%) were characterized by a higher abundance of *Moraxella*, *Haemophilus*, and, which was unique for the study, *Klebsiella*. However, the authors suggest that *Klebsiella* among Chinese children is the second most significant bacteria responsible for community-acquired pneumonia. Zhang L. et al. compared microbiota of the wheezers with that of the control group of children with foreign-body aspiration [47]. Thirty-two children aged 1–3 years with wheezing symptoms, having wheezes at least two times in the past or persistent wheezing for more than one month diagnosed by pediatric pulmonologist, and twenty-three children in the control group had their BAL fluid sample collected during bronchoscopy. In terms of biodiversity, the samples differed only in beta diversity. The most abundant phyla in both groups were Proteobacteria, Firmicutes and Bacteroidetes. The abundance of Proteobacteria phylum was statistically higher in the wheezers group. At the genus level, a higher proportion of *Stenotrophomonas*, *Sphingomonas* and a lower proportion of *Prevotella*, *Neisseria* and *Haemophilus* were obtained in the study group. Yao et al. also assessed the microbiota of wheezers divided in two groups [48]. The criteria for the enrollment in the wheezing groups were the age of 6–36 months, previous wheeze at least three times divided into duration of wheeze to less and more than a month, no history of antibiotic use for at least one week prior to the study and no history of any cardiac and pulmonary diseases. All participants were divided into three groups. The first group included participants with multiple wheezing (*n* = 13), defined by repeated wheezing episodes in a short period of time, the second group included participants with persistent wheezing (*n* = 16), defined as wheezing for more than one month without such symptoms in the past, and the control group (*n* = 19) included children with foreign-body aspiration. The samples of the children’s BAL were obtained during bronchoscopy. As for the previous studies, the most abundant at the phylum level were Proteobacteria, Firmicutes, Bacteroidota and Fucobacteriota. At the genus level, *Phyllobacterium* was the most abundant in both wheezing groups, compared to the controls, and *Sphingomonas* additionally in the multiple wheezing group, compared to the control group. Furthermore, *Neisseria*, *Haemophilus* and *Prevotella* were less abundant in both wheezing groups.

## 4. Summary

The core phyla of the airway bacterial microbiota remain the same between the asthmatics and their healthy peers, and the qualitative differences in phyla seem to be provoked by the changes in the abundance of potentially pathogenic bacteria. In most cases, the asthmatics’ samples from the upper respiratory tract had lower alpha diversity, compared to the healthy groups [20,28]. At the same time, higher richness of bacterial microbiota was observed in some studies [15,25]. Moreover, during the exacerbation of the disease, lower alpha diversity was observed [16]. When comparing various sample types, BAL samples were characterized by higher richness than that of the nasal samples [40,42]. The nasal brushes also had greater bacterial richness than that of the washes [17]. The exhaled breath condensates had lower alpha diversity than that of the oropharyngeal samples [44]. Because of the sampling procedure, the EBC and induced-sputum samples could be contaminated with upper respiratory bacteria, of which researchers should be aware while assessing the outcomes of studies with those sampling methods. No discriminant bacteria were found between the upper and lower tracts of the asthmatic airways, which corresponds to the similarity of the respiratory tract microbiota in the non-asthmatics and healthy population.

No significant differences were found in the asthmatic children’s microbiota that could discriminate it for the outcomes in the adult population. Moreover, the presented studies do not provide sufficient information about the influence of asthma treatment on bacterial microbiome. In one study, the asthma medication intake in the lower-airway samples of the asthmatics was statistically relevant in terms of beta diversity (*n* = 14 vs. 5, *p* = 0.014) [44]. This topic needs further investigation.

The nasal samples with high concentration of Moraxella collected from wheezers either during enrollment or during respiratory infection were found in children with a higher risk of recurrent wheezing and developing asthma in the future [34,35,36]. Interestingly, in some studies, clusters of the *Corynebacterium* genus in the nasal samples were associated with a lower asthma exacerbation/disease-control loss ratio [24,30]. Moreover, the Prevotella genus was inversely associated with asthma [20], and its lower abundance was found in the lower-airway samples from wheezers [47,48].

The mentioned bacteria tend to modify the immunological reaction through the promotion of specific genes in our immune system. The Moraxella-dominant samples were associated with the expression of 32 epithelial genes, mostly mediators of inflammation and apoptosis, whereas in the control group no expression was found [15]. *Streptococcus pneumoniae* in the asthmatics were associated with the arachidonic acid metabolism genes [21]. In Chun et al.’s study, *Corynebacterium* in the nasal samples was negatively correlated with inflammation-promoting genes, and *Actinomyces* in the lower-airway samples were negatively correlated with inflammation genes [42]. *Campylobacter* was found to be positively correlated with several inflammatory cytokines, such as granzyme B, MIP-1β and PD-L1. Additionally, *Haemophilus* was positively correlated with PD-L1, contrary to the negatively associated *Porphyromonas* and *Peptostreptococcus* [43].

## 5. Conclusions

This review provides an overview of the current knowledge about the airway bacterial microbiota of asthmatic children and preschool wheezers. As mentioned above, regardless of their location, the most dominant phyla of the airways are Proteobacteria, Firmicutes, Fusobacterium, Bacteroidetes and Actinobacteria. At the genus level, *Streptococcus*, *Moraxella* and *Haemophilus* were in most cases discriminant, indicating that their role is possibly far more important not only during various respiratory tract infections, but also as bacterial homeostatic and immunomodulatory factors. To assess whether asthma as a disease is characterized by higher bacterial diversity, further studies are needed because of the ambiguous outcomes of the collected data. For now, there is no particular known genus that highly discriminates asthmatics from healthy individuals; therefore, no biological interference in the microbiota of the airways is possible as a preventive intervention to reduce the disease prevalence. As seen in the prospective studies on infant wheezers, there are some visible dysbiosis clusters that may provoke the initiation of persistent wheezing/asthma in the future. Understanding the influence of microbiota on the integrity of the microbiome and immune responses in the airways is crucial for the future pharmacological/environmental implementation of certain strategies against the disease.

## Figures and Tables

**Table 1:** Asthmatics’ upper respiratory tract bacterial microbiome assessment. In all of the presented studies, bacterial analysis was conducted using Operational Taxonomic Units (OTUs). After isolating DNA material, bacterial 16sRNA amplification was conducted for further analysis using sequencing methods. In most cases, alpha and beta diversity and relative abundance of bacterial phyla and genera were assessed in terms of bacterial microbiota.

Reference	Subjects	Specimen	Bacterial Microbiota Findings
Castro-Nallar 2015 [15]	8 asthmatic children and 6 healthy controls aged 6–20 years	Nasal epithelial cells	Ambiguous results in terms of biodiversity among asthmatics’ samples (more species but samples dominated by fewer), mostly dominated by *Moraxella catarrhalis* species; *Escherichia* and *Psychobacter* more abundant in asthmatics’ samples
Depner 2016 [16]	327 throat swabs (16.2% asthmatics) and 68 nasal samples (17.4% asthmatics) of children aged 6–12 years	Throat swabs and nasal samples	Lower alpha and beta diversity of nasal microbiota among asthmatics, higher abundance of *Moraxella* genus
Perez-Losada 2016 [17]	30 asthmatic children aged 6–18 years	Nasal washes and nasal brushes	Nasal brushes are characterized by higher alpha and beta diversity and a more abundant bacterial microbiome
Perez-Losada 2017 [18]	40 children aged 6–18 years	2 nasal washes collected within 5.5–6.5 months apart	*Moraxella, Staphylococcus, Dolosigranulum, Corynebacterium, Prevotella, Steptococcus, Haemophilus, Fusobacterium, Neisseriaceae* were the most abundant genera in consecutive order
Boutin 2017 [19]	27 asthmatic children, 57 with diagnosed cystic fibrosis, 60 healthy children aged 6–12 years	Throat swabs	Higher *Haemophilus* abundance in asthmatic children, compared to other groups, children with CF have less diverse bacterial microbiome than that of asthmatics
Birzele 2017 [20]	84 children including asthmatics aged 6–12 years	Mattress dust samples and nasal swabs	Asthma inversely associated with genus richness both in mattress dust and nasal swabs; inverse relative abundance of *Prevotella* genus in asthmatics nasal swabs
Kim BS 2018 [21]	31 children with asthma, 30 children with asthma in remission, 31 healthy controls; children aged 6–10 years	Nasopharyngeal swabs	The most dominant abundance of Proteobacteria in the control group, asthmatics with higher proportion of Fimicutes and Fusobacteria within the remission group; *Staphylococcus* being most dominant in asthma group; *Streptococcus, Dolosigranulum* and *Corynebacterium* more abundant in asthmatics and remission
Perez-Losada 2018 [22]	163 asthmatic children aged 6–18	Nasal washes	4 main genera detected were *Moraxella*, *Staphylococcus*, *Streptococcus and Haemophilus*
An 2018 [23]	7 asthmatics and 13 children without asthma, aged 5–16 years	Mouth swab, nose swab, throat swab, induced sputum, bronchial fluid	Actinobacteria were the most dominant in the nose and mouth swabs, Fusobacteria in throat swabs and induced sputum
Zhou 2019 [24]	214 asthmatic children aged 5–11 years	Nasal blow samples were taken at randomization point (214) and on the early loss of disease control (105)	*Corynebacterium + Dolosigranulum* cluster dominance was associated with a lower risk of asthma exacerbation, switching to *Moraxella*-dominant cluster involved the highest risk of exacerbation
Espuela-Ortiz 2019 [25]	57 asthmatic children aged 15.6 ± 3.3 years and 57 healthy controls aged 15.0 ± 3.9 years	Saliva samples	Higher alpha diversity among asthmatics and higher abundance of *Streptococcus* and *Veilonella* genus among the group
Chiu 2020 [26]	60 participants, 20 with allergic rhinitis and 18 with asthma (both groups mite-sensitized, aged 4.37 ± 0.45 years), 22 healthy children aged 4.59 ± 0.36 years	Throat swab and stool sample	Lower richness and diversity of airway samples, compared to stool samples; significantly increased *Leptotrichia* and *Selenomonas* genera in asthmatics’ airway samples
Chun 2021 [27]	132 asthmatic children aged 12.5 ± 3.6 years (±1 SD)	Nasal swab of 1 nare and nasal brushing of contralateral	Cat sensitization is associated with lower bacterial diversity; *Corynebacterium* and *S. epidermidis* were associated with the absence of sensitization to cat allergens
Majak 2021 [28]	133 children with chronic rhinosinusitis, including 82 asthmatics, aged 4–8 years	Nasal epithelium samples	Reduced abundance of Patescibacteria with an increase of Actinobacteria and *Staphylococcus* strains in asthmatics’ samples; reduced alpha diversity and more frequent sweets consumption, compared to non-asthmatics
Aydin 2021 [29]	46 asthmatics aged 6–17 years, 61 wheezers <6 years, 39 healthy controls	Nasopharyngeal swabs	Higher Firmicutes abundance among asthmatics, atopic asthmatics were more colonized with *Streptococcus* and *Staphylococcus*
Hou 2022 [30]	33 asthmatics aged 6–17 years, 22 non-asthmatics	Flocked nasopharyngeal swabs	Group characteristic of *Moraxella* microbiome profile for longitudinal asthmatic samples, *Corynebacterium* dominated in stable asthma
Liu 2022 [31]	56 asthmatics aged 3–17 years with recurrent wheeze	Nasopharyngeal swabs, throat swabs, stool samples	In the recovery phase, there has been an increase in *Staphylococcus* and decrease in *Moraxella* abundance

**Table 2:** Wheezers’ upper respiratory tract bacterial assessment. In all of the presented studies, bacterial analysis was conducted using Operational Taxonomic Units (OTUs). After isolating DNA material, bacterial 16sRNA amplification was conducted for further analysis using sequencing methods. In most cases, alpha and beta diversity and relative abundance of bacteria phyla and genera were assessed in terms of bacterial microbiota.

Reference	Subjects	Specimen	Bacterial Microbiota Findings
Powell 2019 [32]	293 newborns enrolled in the study, 98 with complete 24-month follow-up and sequencing data	Oropharyngeal swabs	Colonization with *Neisseria* before age of 1 year is positively associated with a risk of wheezing by the age of 2 years; *Granulicatella* species are negatively associated
Cuthbertson 2019 [33]	109 preschool children with acute wheezing (median age 3.83 years) and 75 non-wheezing controls (median age 3.16 years); children aged 0–16 years	Oropharyngeal swabs	Significant beta diversity change between acute wheezing and 9-month follow-up sample, bronchiolitis diagnosis decreased alpha diversity among acute wheezers
Dumas 2019 [34]	921 children aged <1 year hospitalized with bronchiolitis, followed up to age of 3 years	Nasopharyngeal swabs	Children with *Moraxella* dominant cluster were characterized by higher rate of rhinovirus-induced bronchiolitis, eczema and breathing problems among other groups, lower *Haemophilus* abundance in group with highest rate of RSV-induced bronchiolitis
Mansbach2020 [35]	842 infants hospitalized because of bronchiolitis, followed up to 3 years of age; median age of enrollment 3 months	Nasal swabs	Airway enrichment of *Moraxella* or *Streptococcus* after severe viral infection was associated with a higher risk of developing recurrent wheezes by the age of 3 years and wheezes accompanied by asthma at the age of 4 years
Tang 2021 [36]	289 infants followed up from 2 months to 24 months	Nasopharyngeal mucus sample	*Staphylococcus*-dominant microbiome in the first 6 months of life associated with a higher risk of recurrent wheezing at the age of 3 years, *Moraxella* dominance during wheezing illnesses associated with asthma persisting through later childhood
Aydin 2021 [29]	46 asthmatics aged 6–17 years, 6 wheezers < 6 years, 39 healthy controls	Nasopharyngeal swabs	Higher abundance of Proteobacteria in wheezers, more frequent colonization of Moraxella and Haemophilus, compared to other groups
Song 2022 [37]	Children aged 2–5 years divided into three groups: 16 recurrent wheezers with positive API score, 18 children with upper respiratory tract infection, 36 children in control group without infection	Nasopharyngeal swabs	Recurrent wheezers with dominant Proteobacteria phylum, lower alpha diversity than the healthy control group, *Moraxella catarrhalis* and *Dolosigranulum pigrum* were the most abundant species in all samples

**Table 3:** Asthmatics’ lower respiratory tract bacterial microbiome assessment. In all of the presented studies, bacterial analysis was conducted using Operational Taxonomic Units (OTUs). After isolating DNA material, bacterial 16sRNA amplification was conducted for further analysis using sequencing methods. In most cases, alpha and beta diversity and relative abundance of bacteria phyla and genera were assessed in terms of bacterial microbiota.

Reference	Subjects	Specimen	Bacterial Microbiota Findings
Hilty 2010 [39]	13 asthmatics with severe asthma aged 7–15 years, 7 children as a control group (non-asthmatics)	Bronchoalveolar lavage (BAL)	Higher Proteobacteria and lower Bacteroidetes abundance in the asthmatic group, higher abundance of *Haemophilus* spp. and *Staphylococcus* spp., lower abundance of *Prevotella* spp.
An 2018 [23]	7 asthmatics and 13 children without asthma, aged 5–16 years	Mouth swab, nose swab, throat swab, induced sputum, bronchial fluid	Proteobacteria dominant in bronchial samples, compared to nose and mouth; asthmatics and non-asthmatics did not present any statistically significant differences in phylum abundance
Kloepfer 2018 [40]	36 participants, including 22 asthmatics, median age 3.3 years, interquartile range 3 months–18 years	Nasopharyngeal swabs and bronchoalveolar lavage (BAL)	BALF samples are richer and more diverse in terms of bacterial flora; Streptococcus was the most abundant genus in both sample types; *Prevotella* was more abundant in BALF
Goldman 2018 [41]	31 participants, 15 children with severe asthma (11 years ±4.5), 5 with cystic fibrosis (14.4 years ±2.7), 11 non-asthmatics (5.2 years ±4.1)	Bronchoalveolar lavage (BAL)	13 bacterial genera more abundant in asthmatic patients, compared to non-asthmatics, including *Bacteroides*, *Faecalibacterium* and *Roseburia*
Chun 2020 [42]	27 children with severe persistent asthma (aged 12.6 years ±4.4) and 27 controls (aged 12.6 years ±3.8)	Nasal and bronchial brushing for transcriptome profiling, nasal swabs and BAL for microbiome assessment	*Moraxella* and *Alloiococcus* were hub genera for nasal samples, but not for bronchial; *Corynebacterium* in upper airways and *Actinomyces* in lower airways have a negative correlation with an inflammatory response
Kim YH 2021 [43]	95 children, 67 with stable asthma, 22 with asthma exacerbation, 6 controls, aged 6–15 years	Induced sputum	Proteobacteria more abundant and Actinobacteria less abundant within exacerbations; beta but not alpha diversity changed between exacerbation and stable asthma; *Capnocytophaga* significantly more abundant among the exacerbation group
Bar 2022 [44]	38 children aged 6–18 years, 19 asthmatics, 19 healthy controls	Exhaled breath condensates and nasopharyngeal swabs	Class Gammaproteobacteria and Bacilli were less abundant among asthmatics in breath condensates

**Table 4:** Wheezers’ lower respiratory tract bacterial assessment. in all of the presented studies, bacterial analysis was conducted with using Operational Taxonomic Units (OTUs). After isolating DNA material, bacterial 16sRNA amplification was conducted for further analysis using sequencing methods. In most cases, alpha and beta diversity and relative abundance of bacteria phyla and genera were assessed in terms of bacterial microbiota.

Reference	Subjects	Specimen	Bacterial Microbiota Findings
Robinson 2019 [45]	Children aged 1–6 years, grouped into episodic wheezers (*n* = 14) and multiple-trigger wheezers (*n* = 21)	Bronchoalveolar lavage (BAL)	Higher abundance of *Moraxella* and lower bacterial diversity are associated with lower-airway neutrophilia
Zhang X. 2020 [38]	74 infants <6 months of age with first in their life severe RSV bronchiolitis, follow up until 3 years	Sputum samples collected from trachea by a soft suction catheter	Higher Proteobacteria abundance among children who developed recurrent wheezing; at the genus level, higher abundance of *Haemophilus*, *Moraxella* and *Klebsiella* among the mentioned groups
Wu 2021 [46]	Children up to 24 months old, 35 persistent wheezers and 28 of control group	Bronchoalveolar lavage (BAL)	Higher abundance of *Elizabethkingia* and *Rothia* among wheezers
Zhang L. 2022 [47]	32 children aged 1–3 years with wheezing symptoms, 23 non-wheezers with an aspiration of foreign body	Bronchoalveolar lavage (BAL)	Both groups differed in beta but not alpha diversity; higher Proteobacteria abundance among wheezers
Yao 2022 [48]	Children aged 6–36 months divided into multiple wheezing group *n* = 13, persistent wheezing *n* = 16 and foreign-body aspiration control group *n* = 19	Bronchoalveolar lavage (BAL)	Both wheezing groups’ bacterial diversity was lower, compared to controls; higher abundance of *Phyllobacterium* and lower abundance of *Prevotella*, *Neisseria* and *Haemophilus* in wheezers

## Data Availability

Not applicable.
